# Editorial: Emerging applications of targeted and non-targeted metabolomics to physiology and pathophysiology

**DOI:** 10.3389/fphys.2025.1686757

**Published:** 2025-09-10

**Authors:** Geraldine M. Dowling, Michel Aliani

**Affiliations:** ^1^ Department of Life Sciences, School of Science, Atlantic Technological University, Sligo, Ireland; ^2^ Cameron Forensic Medical Sciences, William Harvey Research Institutes, Barts and The London School of Medicine and Dentistry, Queen Mary University of London, London, United Kingdom; ^3^ Department of Analytical, Environmental and Forensic Sciences, School of Cancer and Pharmaceutical Sciences, Faculty of Life Sciences and Medicine, King’s College London, London, United Kingdom; ^4^ Faculty of Exact Sciences-Toxicology, National University of La Plata, La Plata, Argentina; ^5^ Department of Food and Human Nutritional Sciences, Faculty of Agriculture and Food Sciences, University of Manitoba, Winnipeg, MB, Canada; ^6^ The Division of Neurodegenerative Disorders, St. Boniface Hospital Research Centre, Winnipeg, MB, Canada

**Keywords:** metabolomics, medicine, physiology, pathophysiology, disease

In the ever-changing landscape of biomedical research, metabolomics stands at the forefront of discovery as an “omics” science that captures the dynamic chemical fingerprint of life. By enabling the high-throughput identification and quantification of small molecules (<1500 Da), metabolomics provides a powerful window into the complicated interplay between genetics, environment and physiology. From endogenous compounds like amino acids and lipids to exogenous pollutants and pharmaceuticals, the metabolome reflects both health and disease and leads to generation of new hypotheses.

This Research Topic provide recent contributions to this field which underscores its transformative impact on medicine, physiology and disease modelling.

Zhang et al. explored metabolic disruptions in a rat model of visual fatigue linked to traditional Chinese medicine syndromes, revealing altered sphingolipid pathways. Passadore et al. highlighted amino acid signatures in obese children, linking urinary metabolite patterns to early indicators of insulin resistance and metabolic risk. These findings stress metabolomics’ potential in early diagnosis and personalized interventions.

The utility of metabolomics expands into mechanistic insights. Zhang et al. offered a comprehensive review of lipid droplet-mitochondria interactions, critical in metabolic syndrome and pointed to pharmacological targets that may reshape future obesity treatment. Kenéz et al. connected diet-induced ceramide accumulation to insulin resistance in cattle, an elegant model that reflects similar processes in human metabolic disease, providing a bridge between veterinary science and human health.

In the realm of exercise physiology, Ou et al. and Zhou et al. examined how ischemic preconditioning and enteric-coated bicarbonate impact metabolomic profiles without altering performance, revealing that even interventions with no visible performance boost can profoundly shift underlying biochemistry. Landman et al. showed that simple handgrip exercises and remote ischemic preconditioning reduce key inflammatory markers in cerebral small vessel disease, emphasizing how metabolomics can unravel subtle but clinically significant responses to lifestyle interventions.

On the diagnostic Frontier, Wang et al. demonstrated that serum metabolomics can differentiate renal cell carcinoma from benign tumors with exceptional accuracy. Lin et al. provided insights into high-altitude pulmonary edema by identifying arterial and venous metabolite shifts, paving the way for biomarker discovery in acute physiological stress.

Perhaps most compelling is the emerging picture of how parental genetics even without direct gene transmission, shape offspring metabolism. Zhang et al. showed that maternal and paternal eNOS deficiency leads to distinct phenotypes and metabolomic profiles in offspring, suggesting epigenetic and metabolic inheritance as powerful forces shaping health across generations.

As these studies highlight, metabolomics is more than a research tool, it is a language of life, capturing the nuance of disease progression, therapy response, and physiological adaptation. With mass spectrometry and NMR technologies pushing the boundaries of detection and data integration with genomics and proteomics becoming more refined, the metabolome stands as a critical pillar in systems medicine.

